# Beyond the norm: high protein adherence impacts muscular force and size adaptations

**DOI:** 10.1080/15502783.2025.2550161

**Published:** 2025-08-25

**Authors:** Gabriella Gilbert, Kyle Travis, Antonella V. Schwarz

**Affiliations:** a St. Thomas University, Department of Health Sciences, Miami Gardens, FL, USA; b Liberty University, Department of Health Sciences, Lynchburg, VA, USA; c The Rehabilitation Lab, Miami Shores, FL, USA; d Barry University, Department of Health Promotion and Clinical Practice, Miami Shores, FL, USA

**Keywords:** Hypertrophy, protein, body composition, strength

## Abstract

**Background:**

1.6–2.2 g/kg/day of protein is commonly recommended for skeletal muscle development and strength. However, this range is broad and may obscure critical dose–response effects, with emerging evidence suggesting that intakes below 2.0 g/kg/day could limit adaptations. While many studies report average group outcomes, individual adherence to higher protein targets remains understudied, specifically in real-world training contexts. This study examines whether consistent adherence to >2.0 g/kg/day of protein elicits superior muscle and strength gains compared to lower intakes during an 8-week training program.

**Methods:**

Eight recreationally trained males (22.1 ± 2.0 yrs; 175.2 ± 5.6 cm; 84.2 ± 18.6 kg) completed an 8-week hypertrophy-focused training program (3 days/week). Participants were instructed to maintain a protein intake of 1.6–2.2 g/kg/day, monitored via self-reported food logs. Post-intervention, participants were categorized into low (<2.0 g/kg/day) (*n* = 4) or high (>2.0 g/kg/day) (*n* = 4) adherers based on mean daily intake.

Participants underwent one-repetition maximum (1RM) testing for back squat (BS), bench press (BP), and deadlift (DL) at baseline (T1) and post-intervention (T2), with total strength (TOT) calculated as the sum of all three lifts. Body composition metrics such as body mass (BM), fat free mass (FFM), skeletal muscle estimation (SME), and circumference summation (CirSum) were assessed at T1 and T2 using BodPod, estimation equations, and circumference measurements.

**Results:**

From T1 to T2, the high adherence group showed moderate improvements, with BS (+18.1 kg, +15.2%, g = 0.66), BP (+9.6 kg, +10.6%, g = 0.86), DL (+21.0 kg, +14.9%, g = 0.76), and TOT (+48.8 kg, +13.8%, g = 0.79). In contrast, the low adherence group showed small to moderate increases in maximal strength across all lifts, with BS (+13.6 kg, +9.6%, g = 0.49), BP (+5.7 kg, +5.2%, g = 0.43), DL (+23.8 kg, +15.9%, g = 0.80), and TOT (+43.1 kg, +10.8%, g = 0.63). From T1 to T2, the high adherence group showed trivial to small increases in physiological measures, with BM (+2.8 kg, +3.6%, g = 0.30), FFM (+1.6 kg, +2.4%, g = 0.18), SME (+0.8 kg, +2.8%, g = 0.22), and CirSum (+8.9 cm, +2.0%, g = 0.42). In contrast, the low adherence group showed only trivial increases in physiological measures, with BM (+2.2 kg, +2.7%, g = 0.08), FFM (+1.5 kg, +2.1%, g = 0.12), SME (+0.9 kg, +2.9%, g = 0.11), and CirSum (+2.5 cm, +0.6%, g = 0.04).

**Conclusions:**

Preliminary findings suggest protein intake >2.0 g/kg/day may enhance strength adaptations, though its impact on body composition was minimal. For practitioners, prioritizing higher protein intake could benefit strength outcomes, though longer term research is needed to evaluate hypertrophic effects.

